# Institutional Notes and News

**Published:** 1910-11-19

**Authors:** 


### Institutional Notes and News.

**The Hospital for Women.**

The Hospital for Women in Soho Square, which was founded in 1842, and of which her Majesty Queen Mary is patroness, has just been rebuilt at a cost of £21,000. The new hospital buildings, thoroughly up to date according to the latest requirements of medical science, were opened recently, and there are now sixty-seven patients receiving treatment within the walls. There is also a large out-patient department attached to the hospital, treating on an average about eighty patients daily. Visitors interested in charitable work are cordially invited to inspect the new hospital, and may do so by appointment with the Secretary, Mr. Alfred Hayward. The hospital urgently wants additional income to meet the cost of the upkeep of the new buildings. It is a most worthy charity, being the pioneer of the hospitals for diseases peculiar to women. It treats about 5,000 poor patients every year.

**Doncaster Infirmary.**

We are glad to hear that in the practised opinion of Mr. R. E. Clark, who is Chairman of the board of management at Doncaster, the Infirmary of that city has been able to issue "one of the best reports I have heard during my long connection with the institution." As for the work accomplished during the past twelve months, 514 patients were treated in the wards, and 6,369 out-patients and casualties. As for the balance-sheet, the report states that the total income from all sources amounted to £4,253, while after all expenses had been met, including the necessary expense of investment, a balance of £39 remained in the treasurer's hands. After reading such a good report it is hardly surprising to know that a special compliment was paid by more than one speaker to the ability and powers of Mr. W. E. Lister, the hon. secretary. An interesting annual meeting concluded with a presentation to Dr. Willey, the retiring house surgeon, of which we give an account in the News and Events of the Week column.

**Salford Royal Hospital.**

Mr. Lawrence Pilkington, Chairman of the Board of Management of the Salford Royal Hospital, complained at the recent annual meeting of the somewhat scant attention which his hospital receives from the Saturday and Sunday Fund, some £555, or about one-fifth of the total collected, having fallen to the Royal in recent years. This hospital is confronted with an extension scheme for which £70,000 is required, and of which, we believe, about £47,000 has been given. When completed the need for maintaining the increased accommodation will represent, as Mr. Pilkington aptly pointed out, a serious problem to the board of management. It was, no doubt, because he shared this view that Sir William Mather, who presided, allowed himself some interesting remarks on the possibility of the present Government by-and-by making grants in aid to the hospitals of the country. "They should be grants," Sir William went on, "made in such a way that they will interfere in no way with, nor diminish, the zeal of voluntary work." Sir William Mather, of course, needs no reminding that grants in aid have proved in every case the thin end of the wedge, and anyone who accepts them in the case of hospitals must logically admit the ultimate probability of State control. If at every annual meeting such interesting speeches were made as those at the recent one at Salford, there would be no question of the indifference of the public to them. It is only fair to the Hospital Saturday and Sunday Fund to add that a special committee has been appointed to draft a new scheme of distribution.

**Queen's Hospital for Children.**

We hope that the dinner to take place on behalf of the Queen's Hospital for Children at the Cecil on November 23 will be a success. There is no question of the claim of the hospital to support, for Hackney, where it stands, is one of the poorest districts in London—a district, too, of so wide an area as to embrace Bethnal Green, Hoxton, and Whitechapel. No effort must be spared to make the giving public realise that such places really exist, and are not the nightmare fancies of a Postmaster-General anxious to enliven the Post Office Directory with romantic names. Nevertheless, nearly 2,000 child patients were admitted from these areas last year, 788 of whom were under two years old. In spite of this, the hospital possesses an endowment of £320 only, which, we believe, creates a sad and bad record among institutions of a similar size. The public may with confidence give generous support towards raising the £11,000 that is necessary for the annual income from the facts that a lady almoner is employed to check abuse; that co-operation with other charitable agencies is undertaken; and, finally, that every effort is made to carry the lessons of hygiene, cleanliness, and sanitation learnt in the hospital into the homes of those discharged. The total debt now amounts to £8,500, hence the eloquent appeal of Lord Shaftesbury and Mr. Maurice Rüffer.

**West of England Eye Infirmary.**

The past year, as the Committee's report presented at the recent annual meeting states, in the history of the West of England Eye Infirmary has marked a very important epoch, since the scheme for building a new wing to commemorate the hospital's centenary has been completed. This scheme was started in May 1908, and six weeks ago the formal opening was undertaken by the Right Hon. Earl Fortescue, the Lord-Lieutenant of Devon. It is exceedingly praiseworthy to add that the new buildings were opened free of debt. The block of buildings of which the new wing forms a part is now complete. The architectural crown has been given by it to the plans which began to take the form of bricks and mortar eleven years ago. Turning to the hospital's work during the past year, we find that the daily average number of in-patients has been forty-two, and that 3,710 persons have received the benefit of treatment. It should be added that the medical inspection of school children has brought a total of 396 young patients to the hospital, seventy-six of whom came from Exeter. Referring to them, Mr. H. M. Imbert-Terry, who presided at the annual meeting, said: "If the number of these cases increases to a considerable extent, steps will have to be taken to see that no unduly great labour is thrown upon the medical staff of the infirmary. I wish particularly to praise the work of Mr. S. E. Whitton, the Secretary, and his great zeal and ability. Every member of the Committee will acknowledge that in Mr. Whitton we have an invaluable secretary, who is always ready to give his time and ability to the work. The least thing we can do, therefore, is to pay him a tribute of praise which he has so thoroughly well earned." Dr. Rawson Pickard, M.D., also referred to the school children, pointing out that recommendations were limited in number, and that probably more children would have come to the hospital if they could have been obtained for them. Dr. Pickard added: "It is true that the number of the children from the schools has not been so great as might have been expected, and it is doubtful whether this is satisfactory from a public point of view."

